# *Chlamydia muridarum* Infection of Macrophages Stimulates IL-1*β* Secretion and Cell Death via Activation of Caspase-1 in an RIP3-Independent Manner

**DOI:** 10.1155/2017/1592365

**Published:** 2017-06-04

**Authors:** Lixiang Chen, Xue Liu, Xin Yu, Rongrong Ren, Chao Wang, Rui Zhao, Guangxun Meng, Shun Li, Xiaohui Zhou

**Affiliations:** ^1^Shanghai Public Health Clinical Center, Key Laboratory of Medical Molecular Virology, Ministry of Education and Health, Fudan University, Shanghai 201508, China; ^2^Clinical Laboratory, The Second Affiliated Hospital of Xuzhou Medical University, Xuzhou, Jiangsu 221002, China; ^3^Huazhong Agricultural University, Wuhan 430070, China; ^4^Unit of Innate Immunity, Key Laboratory of Molecular Virology & Immunology, Institut Pasteur of Shanghai, Chinese Academy of Sciences, Shanghai 200031, China

## Abstract

Chlamydiae are Gram-negative bacteria, which replicate exclusively in the infected host cells. Infection of the host cells by Chlamydiae stimulates the innate immune system leading to an inflammatory response, which is manifested not only by secretion of proinflammatory cytokines such as IL-1*β* from monocytes, macrophages, and dendritic cells, but also possibly by cell death mediated by Caspase-1 pyroptosis. RIP3 is a molecular switch that determines the development of necrosis or inflammation. However, the involvement of RIP3 in inflammasome activation by* Chlamydia muridarum* infection has not been clarified. Here, we assessed the role of RIP3 in synergy with Caspase-1 in the induction of IL-1*β* production in BMDM after either LPS/ATP or* Chlamydia muridarum* stimulation. The possibility of pyroptosis and necroptosis interplays and the role of RIP3 in IL-1*β* production during* Chlamydia muridarum* infection in BMDM was investigated as well. The data indicated that RIP3 is involved in NLRP3 inflammasome activation in LPS/ATP-stimulated BMDMs but not in* Chlamydia muridarum* infection. Pyroptosis occurred in BMDM after LPS/ATP stimulation or* Chlamydia muridarum* infection. Moreover, the results also illuminated the important role of the Caspase-1-mediated pyroptosis process which does not involve RIP3. Taken together, these observations may help shed new light on details in inflammatory signaling pathways activated by* Chlamydia muridarum* infection.

## 1. Introduction

Chlamydiae are obligate intracellular Gram-negative bacteria that, each year, are responsible for a considerable number of the eye, genital tract, respiratory tract, vasculature, and joints infections in humans [1, 2]. Despite the threat posed by chlamydial infections to the global public health, the exact molecular mechanism underlying their pathogenesis remains elusive. Since* Chlamydia* replicate only in the infected host cells, the outcome of infection depends on the interactions of* Chlamydia* and the host and the balance in the induction of inflammatory and cell death pathways [3–6].

As a potent pyrogen, IL-1*β* elicits a strong proinflammatory response [7] and plays a critical role in the clearance or* Chlamydia*-associated pathology. IL-1*β* belongs to the IL-1, predominantly proinflammatory, family of cytokines. The production and activation process of the IL-1*β* family of cytokines requires two signals. Activation of NF-*κ*B, resulting from signaling such as TLRs, induces the transcription of IL-1 family cytokines, including pro-IL-1*β* and pro-IL-18. The second signal which is derived from a wide range of cytosolic complexes of host-cell molecules, known as the inflammasome, leads to activation of the protease Caspase-1 necessary for cleavage of the immature form of IL-1 cytokines [8, 9]. One of the most important inflammasomes consists of NLRP3, ASC, and Caspase-1, which is the key executor processing pro-IL-1*β*, pro- IL-18, and so forth into their mature forms [10, 11].

NLRP3 inflammasome could be activated by a variety of inflammatory triggers, including ATP, microbial toxins, and crystalline substances [12, 13]. Some pathogens such as bacteria, fungi, and viruses could also activate NLRP3 inflammasome [14]. For example,* Chlamydia* infections could lead to activation of Caspase-1 in monocytes, macrophages, and dendritic cells and associated IL-1*β* secretion [15–20].

Infection of mice by* Chlamydia muridarum* gives a similar pathology to that shown in humans and for this reason murine models have been employed to study* Chlamydia-*associated pathogenesis [21]. Using this animal model, it was demonstrated that* Chlamydia muridarum* is able to activate Caspase-1 and NLRP3 inflammasome [22–24]. Inflammasome activation results in an inflammatory response and possibly also in cell death attributed to Caspase-1-mediated pyroptosis. It was reported that* Chlamydia* infection could induce necrosis of infected cells; however, the exact underlying mechanisms remain unknown. Delineating the interaction of inflammation pathways and cell death pathways is critical for understanding chlamydial pathogenesis.

Necrosis with programmed cell death features characterizes chlamydiae infection, but the mechanism has not been revealed. Recently, the receptor-interacting protein kinase 3 (RIP3/RIPK3) has emerged as a critical regulator of programmed necrosis referred to as necroptosis [25]. Caspase-1- or Caspase-11-mediated cell death, or pyroptosis, is dependent on the activation of the inflammasome. Few studies demonstrated the interactions among apoptosis, necrosis, and autophagy; however, the interplay between pyroptosis and necroptosis and the secretion of IL-1*β* proinflammatory cytokines as well as the signaling molecules that regulate the two cell death pathways remain obscure.

In the present study, employing a murine model of* Chlamydia muridarum* infection, we investigated the possibility of interplay between pyroptosis and necroptosis in conjunction with the role of RIP3, a key molecule regulating necroptosis pathways and IL-1*β* production in bone marrow derived macrophages (BMDMs).

## 2. Materials and Methods

### 2.1. Mice

Rip3^−/−^ mice were kindly provided by Prof. Xiaodong Wang's group, and Caspase-1^−/−^ mice were purchased from Jackson Laboratory. C57BL/6 mice were purchased from B&K Universal Group Limited (Shanghai, China), and Rip3^−/−^Caspase-1^−/−^ mice were obtained by crossing Rip3^−/−^ and Caspase-1^−/−^ mice. Mice were kept under specific pathogen-free conditions at the Animal Center, Shanghai Public Health Clinical Center. All animal-related procedures were performed according to the Institutional Animal Care and Use Committee (IACUS).

### 2.2. Reagents

The materials used were as follows:* Chlamydia muridarum* (*C. muridarum*,* Chlamydia* mouse strain Nigg II; ATCC, USA); Iscove's modified Dulbecco's medium (IMDM, Gibco); fetal bovine serum (FBS, Gibco, USA); cycloheximide (Sigma-Aldrich, USA); macrophage colony-stimulating factor (m-CSF, PeproTech, USA); penicillin-streptomycin (Gibco, USA); nonessential amino acid (Gibco, USA); LPS (Sigma-Aldrich, USA, cat. number L3012); ATP (Sigma-Aldrich, USA); anti-mouse Caspase-1p20 (Adipogen, USA); anti-rabbit IL-1beta (Sigma-Aldrich, USA); anti-mouse *β*-actin (Abcam, USA); protease inhibitor (PI, Roche, Switzerland); phosphatase inhibitors (PhosSTOP, Roche, Switzerland); Annexin V-APC (BD, USA); Annexin V-FITC/propidium iodide (PI) assay kit (KeyGen, China); mouse IL-1beta ELISA Ready-Set-Go kit (eBioscience, USA); FAM-FLICA-Caspase-1 kit (AbD Serotec, Switzerland); CytoTox96 Nonradioactive Cytotoxicity Assay (Promega, USA); 2x SDS loading buffer (Tris, 1.21 g; SDS, 4 g; *β*-mercaptoethanol, 10 ml; glycerol, 20 ml; bromophenol blue, 0.2 g; double distilled water to 1000 ml); lysis buffer (1000 ml: 2x SDS loading buffer, 840 *μ*l; *β*-mercaptoethanol, 100 *μ*l; PI, 50x, 20 *μ*l; PS, 25x, 40 *μ*l).

### 2.3. Chlamydial Propagation

BGMK cells were kindly gifted by the University of South China and used for* C. muridarum* propagation. BGMK cells were infected by* C. muridarum*, and EBs were purified by gradient centrifugation. The infectivity titers were determined by immunofluorescence quantity analysis. The inclusion-forming units per milliliter were 1 × 10^9^. Cells were cultured in IMDM with 10% FBS and 1% penicillin-streptomycin and maintained in an incubator at 37°C and 5% CO_2_. Logarithmically growing cells were used for experiments.

### 2.4. Macrophage Differentiation and Stimulation

Bone marrow cells were harvested from 8- to 12-week-old mice, and they were differentiated into macrophages (bone marrow derived macrophages, BMDMs) by culturing in IMDM containing 40 ng/ml M-CSF, 10% FBS, 1% nonessential amino acid, and 1% penicillin-streptomycin. After six days, BMDMs were seeded into six-well cell culture plates, and on the following day, LPS (50 ng/ml) was added and cultured for 6 hours. At the last thirty minutes of 6 hours, 0.5 M ATP was added. For* Chlamydia* infection, BMDMs were infected by 10 Multiplicity of Infection (MOI)* Chlamydia muridarum* for either 12 or 24 hours.

### 2.5. Western Blotting

Cell supernatants were collected and concentrated by methanol and chloroform. The concentrated precipitation and cells were washed and lysed in lysis buffer. Samples were denatured in loading buffer and boiled at 100°C for 10 minutes. Proteins separated by SDS-PAGE were transferred to nitrocellulose membranes and immunoblotted with primary antibodies against Caspase-1, IL-1*β*, and *β*-actin, followed by HRP-conjugated secondary antibodies. ECL reagent was used for western blot development.

### 2.6. Flow Cytometry and Cell Death Assay

BMDMs, preinfected with* C. muridarum* or stimulated with LPS and ATP, were stained with Caspase-1 FLICA, and PI or Annexin V for cell death analysis on an LSR II using FlowJo software (BD Biosciences).

### 2.7. Cytokine Analysis

Mouse IL-1*β* Ready-Set-Go ELISA kit was used to detect the concentrations of IL-1*β* cytokines in the cell supernatants.

### 2.8. Lactate Dehydrogenase (LDH) Release Assay

The quantity of LDH released into the culture medium was detected for evaluating cell membrane disruption using the CytoTox96 Nonradioactive Cytotoxicity Assay kit in accordance with the manufacturer's instructions.

### 2.9. Statistics

Data were analyzed by GraphPad Prism 5.0 software and presented as mean ± standard deviation. Student's *t*-test was used to determine statistical significance. The figures are shown by combining the values of three independent experiments. *p* < 0.05 was considered statistically significant (values = mean ± SE; ^*∗*^*p* < 0.05, ^*∗∗*^*p* < 0.01, and ^*∗∗∗*^*p* < 0.001).

## 3. Results

### 3.1. NLRP3 Inflammasome Activation Induced by LPS/ATP Stimulation in Macrophage

**Figure 1:**
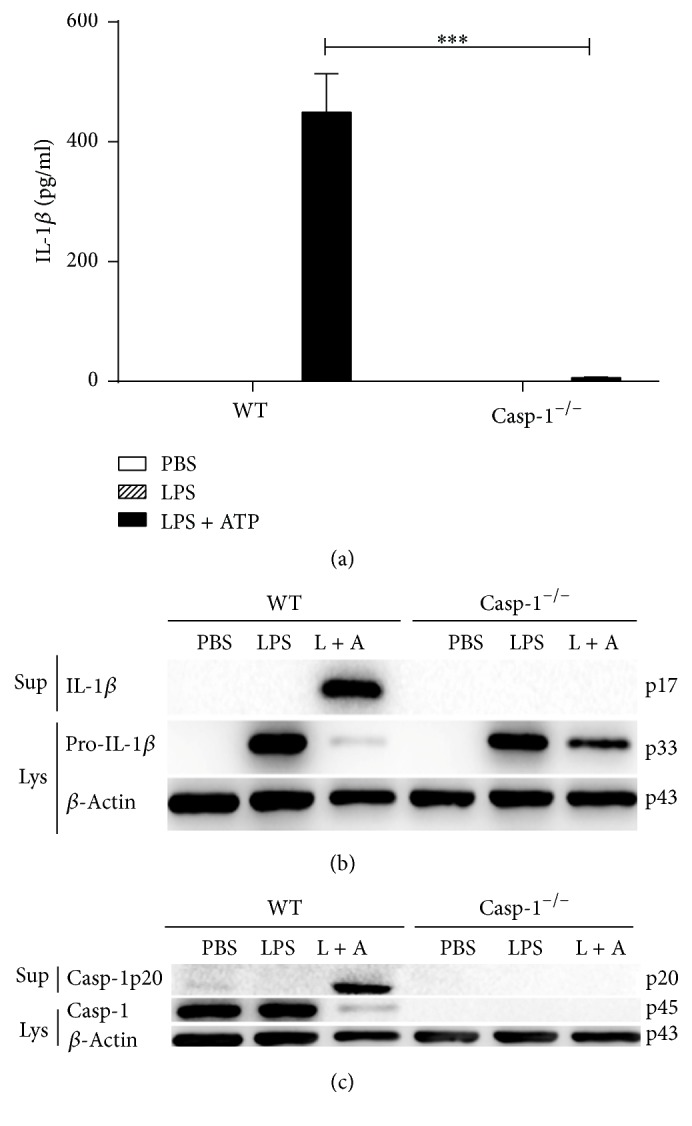
The activation of NLRP3 inflammasome induced by the LPS/ATP treatment. WT, Caspase-1^−/−^ BMDMs were stimulated by LPS/ATP and supernatants were analyzed for the secretion of IL-1*β* by ELISA (a). Western blot of both cell supernatants and lysates was performed for the detection of IL-1*β* (b) and Caspase-1 (c). The figure is shown by combining the values of three independent experiments; values = mean ± SE; ^*∗∗∗*^*p* < 0.001.

It is well established that NLRP3 inflammasome is activated following LPS/ATP treatment [21]. To confirm whether the IL-1*β* secretion is dependent on Caspase-1 activation, the bone marrow cells obtained from C57BL/6 (wild type, WT) mice or Caspase-1^−/−^ mice (C57BL/6 background) were differentiated into bone marrow derived macrophages (BMDMs) using M-CSF. BMDMs were then treated with either PBS, LPS, or LPS/ATP. As expected, IL-1*β* was detected in culture supernatants of WT but not of Casp-1^−/−^ BMDMs following LPS/ATP stimulation (Figure 1(a)). To further confirm LPS/ATP-mediated activation of NLRP3 inflammasome, the cleavage of IL-1*β* and Caspase-1 was examined by western blot immunoanalysis. The results show the presence of a mature form of IL-1*β* and Caspase-1 in supernatants of WT BMDMs following LPS/ATP stimulation (Figures 1(b) and 1(c), resp.).

### 3.2. The Role of Caspase-1 in the Release of LDH in LPS/ATP-Stimulated BMDMs

**Figure 2:**
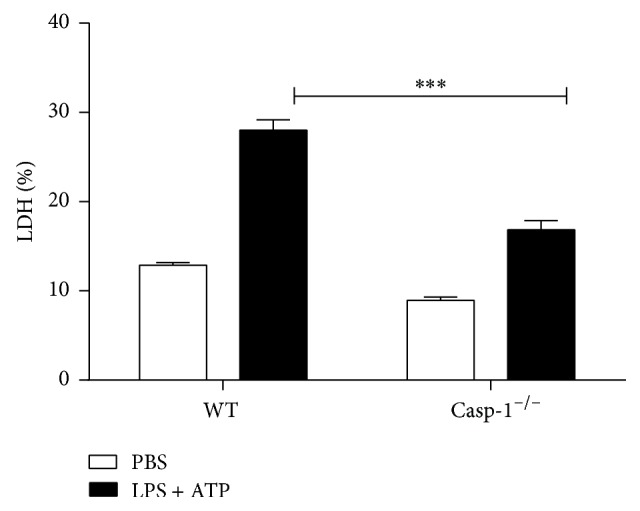
The release of LDH induced by LPS/ATP treatment. The figure is shown by combining the values of three independent experiments; values = mean ± SE; ^*∗∗∗*^*p* < 0.001.

We demonstrated activation of NLRP3 inflammasome following LPS/ATP stimulation of BMDMs. Pyroptosis is a form of inflammatory cell death where activation of NLRP3 inflammasome results in the loss of cell membrane integrity and release of lactate dehydrogenase (LDH) that is normally maintained within the cell cytosol. We, therefore, compared whether LPS/ATP stimulation of WT or Caspase-1^−/−^ BMDMs leads to cell death manifested by the release of LDH. Our results show a significant increase in the release of LDH from LPS/ATP-stimulated WT BMDMs compared to Caspase-1^−/−^ BMDM, suggesting occurrence of pyroptosis (Figure 2).

### 3.3. The Role of RIP3 and Caspase-1 in the Activation of NLRP3 Inflammasome Induced by LPS/ATP Stimulation

**Figure 3:**
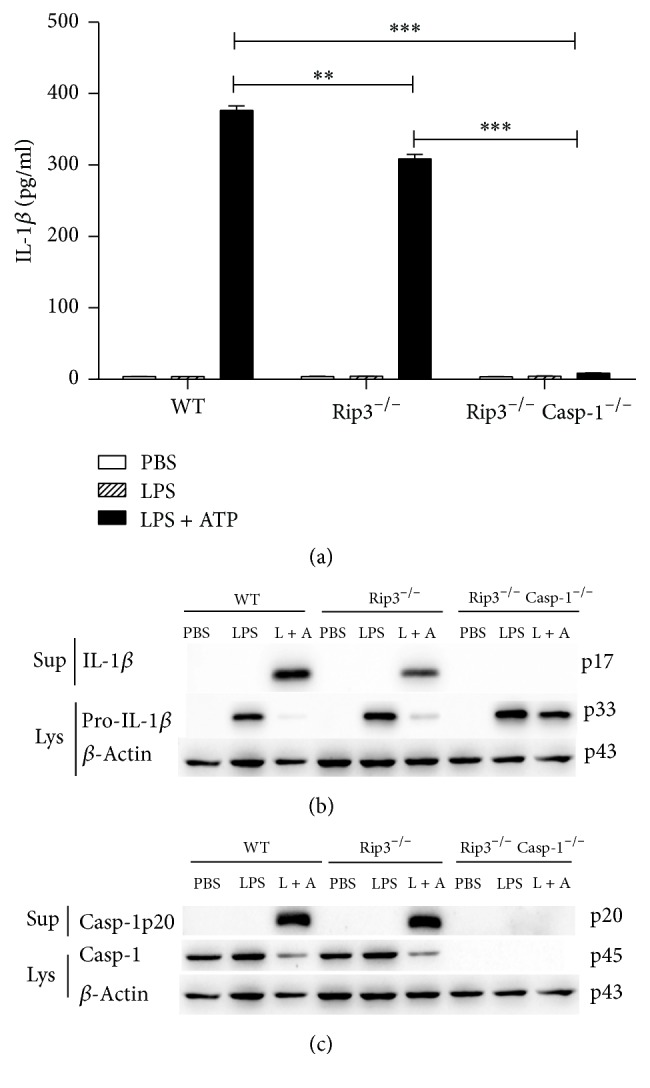
The role of RIP3 in the activation of NLRP3 inflammasome induced by the LPS/ATP treatment. WT, RIP3^−/−^, RIP3^−/−^Caspase-1^−/−^ BMDMs stimulated by LPS/ATP and supernatants were analyzed for the secretion of IL-1*β* by ELISA (a). Western blot of both cell supernatants and lysates was performed for the detection of IL-1*β* (b) and Caspase-1 (c). The figure is shown by combining the values of three independent experiments; values = mean ± SE; ^*∗∗*^*p* < 0.01, ^*∗∗∗*^*p* < 0.001.

**Figure 4:**
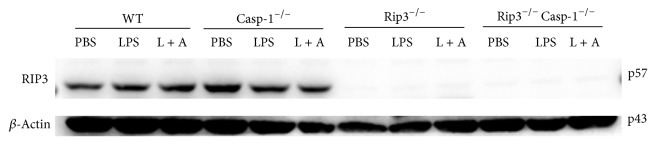
The RIP3 expression levels in WT, Casp-1^−/−^, RIP3^−/−^, RIP3^−/−^Caspase-1^−/−^ BMDMs.

RIP3 is an important regulator of programmed necrosis, which can also promote inflammation independent of its pronecrotic activity. To obtain insights into the involvement of RIP3 in the activation of canonical NLRP3 inflammasome, we employed BMDMs generated from RIP3^−/−^, RIP3^−/−^Caspase-1^−/−^, or WT mice followed by stimulation with LPS/ATP or PBS as a control. Secretion of IL-1*β* to culture supernatants and the presence of the cleaved forms of IL-1*β* and Caspase-1 were examined in culture supernatants and cell lysates. As shown in Figure 3(a), compared with WT BMDMs, the secretion of IL-1*β* and the cleavage of IL-1*β* and Caspase-1 were reduced in RIP3^−/−^ BMDMs, suggesting RIP3 involvement in this process. Moreover, knockout of both RIP3 and Caspase-1 abrogated NLRP3 inflammasome activation after stimulation, underscoring the significant role of Caspase-1 in NLRP3 inflammasome activation (Figures 3(b) and 3(c)). The expression level of Rip3 in WT, Casp-1^−/−^, Rip3^−/−^, and Rip3^−/−^Casp-1^−/−^ BMDMs which were treated with either PBS, LPS, or LPS/ATP was checked by southern blot. The results showed Rip3 in WT and Casp-1^−/−^ BMDMs treated with PBS, LPS, or LPS/ATP having similar expression levels (Figure 4). As we expected, Rip3 expression was not detected in both Rip3^−/−^ and Rip3^−/−^Casp-1^−/−^ BMDMs (Figure 4).

### 3.4. The Role of RIP3 in the Release of LDH Induced by LPS/ATP Stimulation

**Figure 5:**
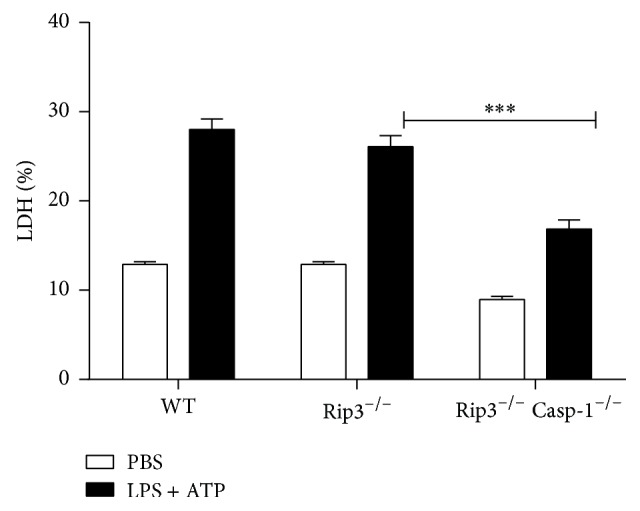
The role of RIP3 in the release of LDH induced by LPS/ATP treatment. The figure is shown by combining the values of three independent experiments; values = mean ± SE; ^*∗∗∗*^*p* < 0.001.

To study the role of RIP3 in BMDMs death following LPS/ATP stimulation, we compared the release of LDH from RIP3^−/−^, RIP3^−/−^Casp-1^−/−^, and WT BMDMs. The results demonstrated similar levels of LDH released from LPS/ATP-stimulated RIP3^−/−^ and WT BMDMs, suggesting that RIP3 is not involved in the cell death induced by LPS/ATP (Figure 5). However, significantly different levels of LDH were released from RIP3^−/−^ and RIP3^−/−^Caspase-1^−/−^ BMDMs, confirming the important role in the cell death process.

### 3.5. The Activation of NLRP3 Inflammasome Induced by* C. muridarum*

**Figure 6:**
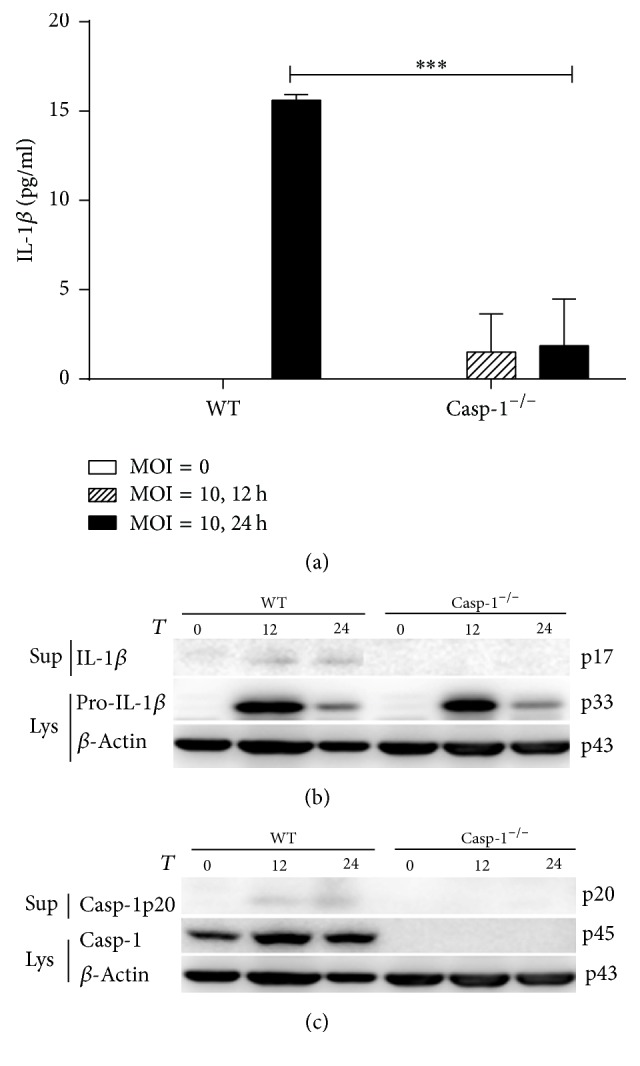
The activation of NLRP3 inflammasome induced by* C. muridarum*. WT, Caspase-1^−/−^ BMDMs were infected by* C. muridarum* (MOI = 10) for 12 h and 24 h. Supernatants were analyzed for the secretion of IL-1*β* by ELISA (a). Western blot of both cell supernatants and lysates was performed for the detection of IL-1*β* (b) and Caspase-1 (c). The figure is shown by combining the values of three independent experiments; values = mean ± SE; ^*∗∗∗*^*p* < 0.001.

We further determined whether* C. muridarum* infected BMDMs secrete IL-1*β* and whether this process requires inflammasome-dependent Caspase-1 activation. BMDMs from Caspase-1^−/−^ and WT mice were infected with* C. muridarum* (MOI = 10) for either 12 hours or 24 hours. We detected IL-1*β* secretion in culture supernatants (Figure 6(a)) and the cleavage of IL-1*β* (Figure 6(b)) and Caspase-1 (Figure 6(c)) both in WT BMDM. However, in Caspase-1^−/−^ BMDM, almost no IL-1*β* secretion and cleavage of IL-1*β* and Caspase-1 were detected. These results suggest that Caspase-1 plays a significant role in the activation of NLRP3 inflammasome during* C. muridarum* infection.

### 3.6. The Role of RIP3 in the Activation of NLRP3 Inflammasome Induced by* C. muridarum*

**Figure 7:**
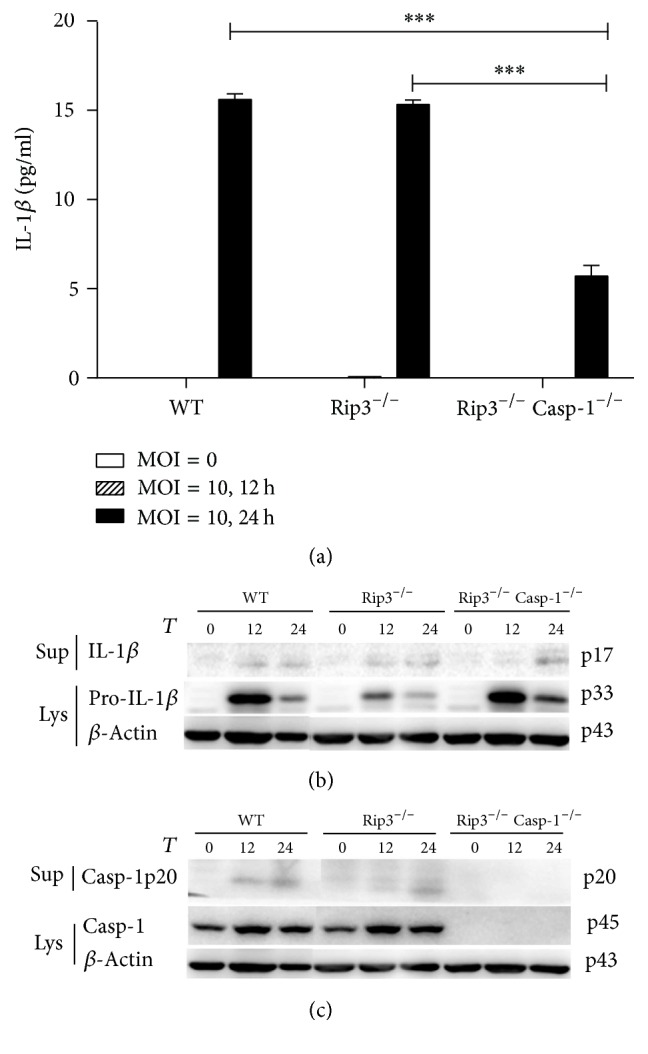
*C. muridarum *induced the activation of NLRP3 inflammasome independent of RIP3. WT, RIP3^−/−^, RIP3^−/−^Caspase-1^−/−^ BMDMs were infected by* C. muridarum *(MOI = 10) for 12 h and 24 h. Supernatants were analyzed for the secretion of IL-1*β* by ELISA (a). Western blot of both supernatants and lysates was performed for the detection of IL-1*β* (b) and Caspase-1 (c). The figure is shown by combining the values of three independent experiments; values = mean ± SE; ^*∗∗∗*^*p* < 0.001.

Our previous results showed that RIP3 might be involved in the NLRP3 inflammasome activation during LPS/ATP stimulation. We thus examined whether RIP3 could regulate NLRP3 inflammasome activation induced by* C. muridarum*. Therefore, we infected BMDMs from WT, RIP3^−/−^, and RIP3^−/−^Caspase-1^−/−^ mice with* C. muridarum* (MOI = 10). The results showed that there were no significant differences in IL-1*β* secretion and Caspase-1 cleavage between RIP3^−/−^ and WT BMDMs (Figure 7(a)). In RIP3^−/−^Caspase-1^−/−^ BMDMs, nearly no IL-1*β* secretion and cleavage of IL-1*β* and Caspase-1 were detected (Figures 7(b) and 7(c)).

### 3.7. The Role of RIP3 and Caspase-1 in Cell Death Induced by* C. muridarum*

**Figure 8:**
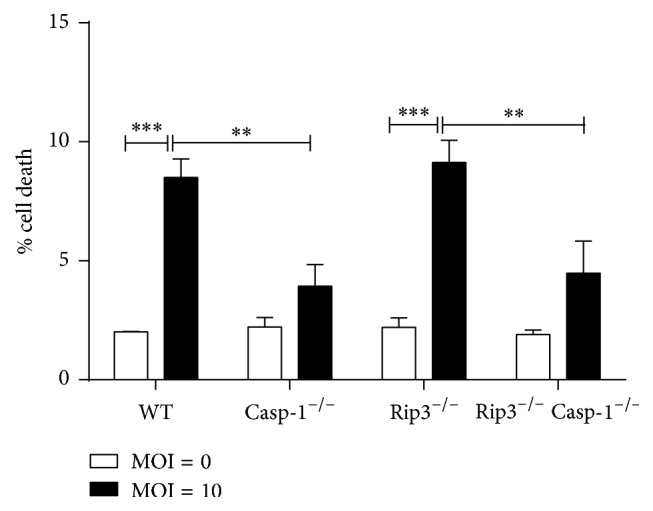
The role of RIP3 in cell death induced by* C. muridarum*. WT, Caspase-1^−/−^, RIP3^−/−^, RIP3^−/−^Caspase-1^−/−^ BMDMs were infected with* C. muridarum *(MOI = 10) for 12 h and 24 h and cell death was analyzed by flow cytometry. The figure is shown by combining the values of three independent experiments; values = mean ± SE; ^*∗∗*^*p* < 0.01, ^*∗∗∗*^*p* < 0.001.

To examine whether RIP3 and Caspase-1 are involved in cell death induced by* C. muridarum*, we infected BMDMs from either WT, Caspase-1^−/−^, RIP3^−/−^, or RIP3^−/−^Caspase-1^−/−^ mice with* C. muridarum *(MOI = 10). Flow cytometry was used for detection of cell death. The results (Figure 8) showed that cell death was increased after* C. muridarum* infection in the four different sets of BMDMs compared to their corresponding control groups without* C. muridarum* infection. Compared to the wild type BMDMs, in Caspase-1^−/−^ BMDMs, the ratio of cell death was reduced after* C. muridarum* infection. However, in the RIP3^−/−^ BMDMs, the ratio of cell death showed no significant differences compared with WT BMDMs after* C. muridarum* infection. The Caspase-1^−/−^ RIP3^−/−^ BMDMs showed almost similar results to Caspase-1^−/−^ BMDMs compared with WT BMDMs after* C. muridarum *infection.

## 4. Discussion

NLRP3 inflammasome can be activated by bacterial, fungal, viral, and other agents such as ATP [26]. Previous studies demonstrated that canonical NLRP3 inflammasome activated by LPS/ATP in vitro induces secretion of IL-1*β* and pyroptosis [27]. We utilized LPS plus ATP, as a PAMP and a DAMP (damage associated molecular pattern), to stimulate either Caspase-1 deficient, RIP3 deficient, or Caspase-1/RIP3 double deficient and WT BMDMs. Stimulation of WT BMDMs with LPS/ATP led to IL-1*β* secretion and Caspase-1 cleavage indicating activation of the NLRP3 inflammasome and IL-1*β* cleavage mediated by Caspase-1. In contrast, Caspase-1 deficient and Caspase-1/RIP3 double deficient BMDMs were unable to secrete IL-1*β* and consequently did not cause Caspase-1 cleavage. However, RIP3 deficient BMDMs were proficient in secretion of IL-1*β* and in the cleavage of Caspase-1 albeit at reduced rates compared with WT BMDMs. These results prompted us to examine the role of RIP3 in the process of NLRP3 inflammasome activation following LPS/ATP stimulation of BMDMs. Since LPS activates CD14/TLR4 and ATP is known to stimulate the nucleotide receptor P2X_7_ [28], we hypothesized that RIP3 may take part in the signaling pathways downstream of TLR4 and P2X_7_.

Caspase-1 is required for induction of pyroptosis and production of the proinflammatory cytokine IL-1*β* [29]. Therefore, employing WT and Caspase deficient BMDMs, we investigated the involvement of Caspase-1 in the regulation of cell death following stimulation of BMDMs with LPS/ATP. We demonstrated that Caspase-1 deficient or Caspase-1/RIP3 double deficient BMDMs stimulated with LPS/ATP released significantly lower levels of LDH compared to WT controls. The dominant role of Caspase-1 in the release of LDH is further underscored by the fact that RIP3 deficient BMDMs were able to release the same level of LDH as WT controls. Together, these observations suggest induction of pyroptosis following LPS/ATP stimulation of BMDMs and illuminate the important role Caspase-1 and lack of involvement of RIP3 in this process.

It has been demonstrated that infection of monocytes and macrophages by some strains of* Chlamydia*, including* Chlamydia trachomatis*,* Chlamydia muridarum*, and* Chlamydia psittaci*, leads to IL-1*β* secretion consequent to the activation of Caspase-1 [15]. Studies also showed that RIP3 can promote inflammation independent of its pronecrotic activity. However, it is not clear whether, in* Chlamydia muridarum *infected immune cells, RIP3, alone or synergistically with Caspase-1, is required for IL-1*β* secretion and is involved in pyroptosis. It has been reported that macrophages mediate crucial innate immune responses via Caspase-1-dependent processing and secretion of IL-1*β* and IL-18 [30]. To obtain some insights, we used BMDMs with the different backgrounds and studied the impact of* Chlamydia muridarum *infection on IL-1*β* secretion and Caspase-1 cleavage. We showed that* Chlamydia muridarum* infection of WT BMDMs leads to IL-1*β* secretion and Caspase-1 cleavage, indicating NLRP3 inflammasome activation. In contrast, Caspase-1 deficient and Caspase-1/RIP3 double deficient BMDMs infected with* Chlamydia muridarum* were incapable of IL-1*β* secretion and Caspase-1 cleavage, which was in accordance with our results using LPS/ATP stimulation. However, there were no significant differences in IL-1*β* secretion and Caspase-1 cleavage between RIP3^−/−^ and WT BMDMs after* Chlamydia muridarum *infection. This result is inconsistent with RIP3^−/−^ BMDMs stimulated with LPS/ATP. We presume that the downstream signaling pathways activated by LPS/ATP stimulation may vary from those engaged by* Chlamydia muridarum. *Moreover, in* Chlamydia muridarum* infection, RIP3 may not be involved in NLRP3 inflammasome activation, which is in contrast to what we observed following LPS/ATP stimulation of BMDMs. Currently, four distinct cellular death programs have been identified, namely, apoptosis, necroptosis, autophagy, and pyroptosis [31, 32].* Chlamydia* infected cells were reported to be resistant to numerous experimental apoptotic stimuli, exhibiting inhibition of Caspase activation and blockage of cytochrome C release from mitochondria [33, 34]. And it has been reported that* Chlamydia *lymphogranuloma venereum can strongly induce autophagy in the middle of the chlamydial developmental cycle (24 h after infection), suggesting that autophagy is activated primarily in response to the metabolic stress consequent to chlamydial replication [35]. Miao et al. [30] reported that Caspase-1-induced pyroptosis is an innate immune effector mechanism against intracellular bacteria. Employing the flow cytometry analyses, we detected pyroptosis in BMDMs either infected with* Chlamydia muridarum* or stimulated with LPS/ATP. Our results provide evidence for an important role of Caspase-1 in pyroptosis and a dispensary role of RIP3 in pyroptosis of BMDMs either infected with* Chlamydia muridarum* or stimulated with LPS/ATP.

In this study, we assessed the role of RIP3 and Caspase-1 and their synergistic function in IL-1*β* secretion and Caspase-1 cleavage in BMDMs employing two independent systems: LPS/ATP stimulation and* Chlamydia muridarum *infection. The exact mechanisms or downstream signaling pathways, engaged by* Chlamydia muridarum* or by LPS/ATP and required for activation of inflammasome, will require further studies. Our studies however provide evidence for the differential role of RIP3 in NLRP3 inflammasome activation in BMDMs following LPS/ATP stimulation or* Chlamydia muridarum* infection and as such may help to shed new light on details in inflammatory signaling pathways induced by* Chlamydia muridarum* infection.
